# Extensive sphenoid chordoma mimicking a prolactinoma

**DOI:** 10.11604/pamj.2019.33.138.12897

**Published:** 2019-06-24

**Authors:** Mounira El Euch, Olfa Hentati, Madiha Mahfoudhi, Wifak Bani, Fethi Ben Hamida, Fatima Jaziri, Khaoula Ben Abdelghani, Sami Turki, Taieb Ben Abdallah

**Affiliations:** 1 Internal Medicine Department « A », University of Tunis El Manar, Tunis, Tunisia; 2 Research Laboratory of Kidney Diseases (LR00SP01), Charles Nicolle Hospital, Tunis, Tunisia

**Keywords:** Prolactinoma, chordoma, hypopituitarism

## Abstract

The chordoma is a benign cartilaginous tumor whose sphenoidale localization is exceptional. This tumor has considerable difficulties of both diagnosis and treatment. We report the observation of a Tunisian adult who presented features of hypopituitarism set wrongly on account of a prolactinoma.

## Introduction

Chordomas are rare slow growing tumours which diagnosis is very difficult. In this report we describe a patient who presented features of intracranial hypertension, and was thought to have a prolactinoma, which in fact turned out to be an intrasellar chordoma.

## Patient and observation

**Figure 1:**
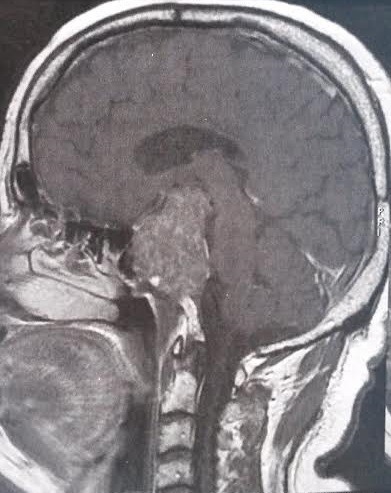
MRI of the brain showing an expansive intrasellar process of 3.7cm

A 60 years old man presented with headache and visual field. MRI of the brain showed an expansive intrasellar process of 3.7cm (Figure 1). The diagnosis of prolactinoma was initially brought with a high rate of prolactinemia and he was treated by bromocriptine with favourable response clinically. Ten years later, he presented acute insufficient adrenal after a surgery of cholecystectomy. Hormonal explorations had objectified a gonadotropic hypopituitarism (luteinising hormone = 0.7mU/ml; testosterone = 0.17; follicle stimulating hormone = 4.3pg/ml; cortisol = 42pg/ml; thyroid stimulating hormone = 1 mIU/l; prolactin = 700ng/ml). He was treated by hemisuccinate of hydrocortisone relayed by cortef with good evolution. Six months later, he presented with headache with right hemiplegia. A MRI scan showed recurrence of tumor with right parasellar extension, compressing the optic chiasm and invading cavernous sinus. He had trans-sphenoidal resection of the tumour. Histology concluded to sphenoid chordoma. The follow up was made by the paralysis of nerve VI. Adjuvant radiotherapy was necessary with a favourable response clinically and radiogically.

## Discussion

Pseudohyperprolactinemia is a frequent situation which pose a diagnostic challenge. Only four cases of pseudohyperprolactinoma have been reported previously where chordoma was associated with increased prolactin values [1-3]. The mechanism underlying the higher level of serum prolactin is not fully understood. Pituitary talk compression syndrome was proposed to explain this phenomenon [3]. Chordomas are hardly distinguished from pituitary adenomas. The presenting symptoms depend both on the location and the size of the tumour. Patients who have chordoma involving the skull base usually present with headache [4]. The endoscopic endonasal surgery is a safe, minimally invasive and efficient procedure for skull base tumor [5]. Chordomas can be locally aggressive and recur. Treatment options include extensive surgical esection, external beam radiotherapy or a combination of both [6].

## Conclusion

Essentially characterized by its great diversity, tumor pathology of the sphenoid is also distinguished by the relative difficulty of radiological diagnosis because of the variable volume of the lesions, tumour extension type as well as the poor evocative clinical features. This case is unusual and shows that chordomas can mimic pituitary adenomas. We have to recognize this disorder not only for appropriate treatment but also for prognostic.

## Competing interests

The authors declare no competing interests.
